# Predictive Power of Air Travel and Socio-Economic Data for Early Pandemic Spread

**DOI:** 10.1371/journal.pone.0012763

**Published:** 2010-09-15

**Authors:** Parviez Hosseini, Susanne H. Sokolow, Kurt J. Vandegrift, A. Marm Kilpatrick, Peter Daszak

**Affiliations:** 1 EcoHealth Alliance (formerly Wildlife Trust), New York, New York, United States of America; 2 University of California Santa Cruz, Santa Cruz, California, United States of America; Yale University, United States of America

## Abstract

**Background:**

Controlling the pandemic spread of newly emerging diseases requires rapid, targeted allocation of limited resources among nations. Critical, early control steps would be greatly enhanced if the key risk factors can be identified that accurately predict early disease spread immediately after emergence.

**Methodology/Principal Findings:**

Here, we examine the role of travel, trade, and national healthcare resources in predicting the emergence and initial spread of 2009 A/H1N1 influenza. We find that incorporating national healthcare resource data into our analyses allowed a much greater capacity to predict the international spread of this virus. In countries with lower healthcare resources, the reporting of 2009 A/H1N1 cases was significantly delayed, likely reflecting a lower capacity for testing and reporting, as well as other socio-political issues. We also report substantial international trade in live swine and poultry in the decade preceding the pandemic which may have contributed to the emergence and mixed genotype of this pandemic strain. However, the lack of knowledge of recent evolution of each H1N1 viral gene segment precludes the use of this approach to determine viral origins.

**Conclusions/Significance:**

We conclude that strategies to prevent pandemic influenza virus emergence and spread in the future should include: 1) enhanced surveillance for strains resulting from reassortment in traded livestock; 2) rapid deployment of control measures in the initial spreading phase to countries where travel data predict the pathogen will reach and to countries where lower healthcare resources will likely cause delays in reporting. Our results highlight the benefits, for all parties, when higher income countries provide additional healthcare resources for lower income countries, particularly those that have high air traffic volumes. In particular, international authorities should prioritize aid to those poorest countries where both the risk of emerging infectious diseases and air traffic volume is highest. This strategy will result in earlier detection of pathogens and a reduction in the impact of future pandemics.

## Introduction

Predicting the origin and emergence of new diseases is critical to preventing and controlling them [1], [2]. In particular, if the early spread of a newly emerging pathogen can be predicted and curtailed before it becomes pandemic, its impact on public health and global economies may be much reduced [3], [4], [5], [6]. In March and April of 2009, a novel H1N1 influenza A virus (2009 A/H1N1) with gene segments from humans, swine, and birds led to the first pandemic of influenza in forty years [7], [8], [9], [10]. Current evidence points to a Mexican origin for the initial human-to-human transmission of this virus, although preliminary genetic analyses suggest the virus has an older and highly-mixed lineage [8]. The virus' lineage and rapid spread suggest that global trade and travel may have played an important role in its early emergence [7], [8]. Here, we attempt to elucidate how these factors may relate to the emergence and spread of this newly detected virus.

One unresolved question is to what degree does a country's development affects its ability to detect and respond to an emerging disease in a timely manner? Development may affect spending on healthcare infrastructure, and particularly, spending on the high cost, intensive public health surveillance needed during the early stages of a pandemic [11], [12], [13]. Socioeconomic factors will also likely affect individuals' abilities or desire to seek diagnosis or treatment, and a country's capacity to test and identify pathogens. Here, we analyze socio-economic and travel data to understand the initial spread of this virus. We focus on the early stages of the epidemic, when travel from Mexico was likely to be the dominant mode of viral spread. Finally, we examine poultry and swine trade data prior to the 2009 A/H1N1 pandemic to add to our understanding the processes that led to the emergence of this virus.

## Results

**Figure 1:**
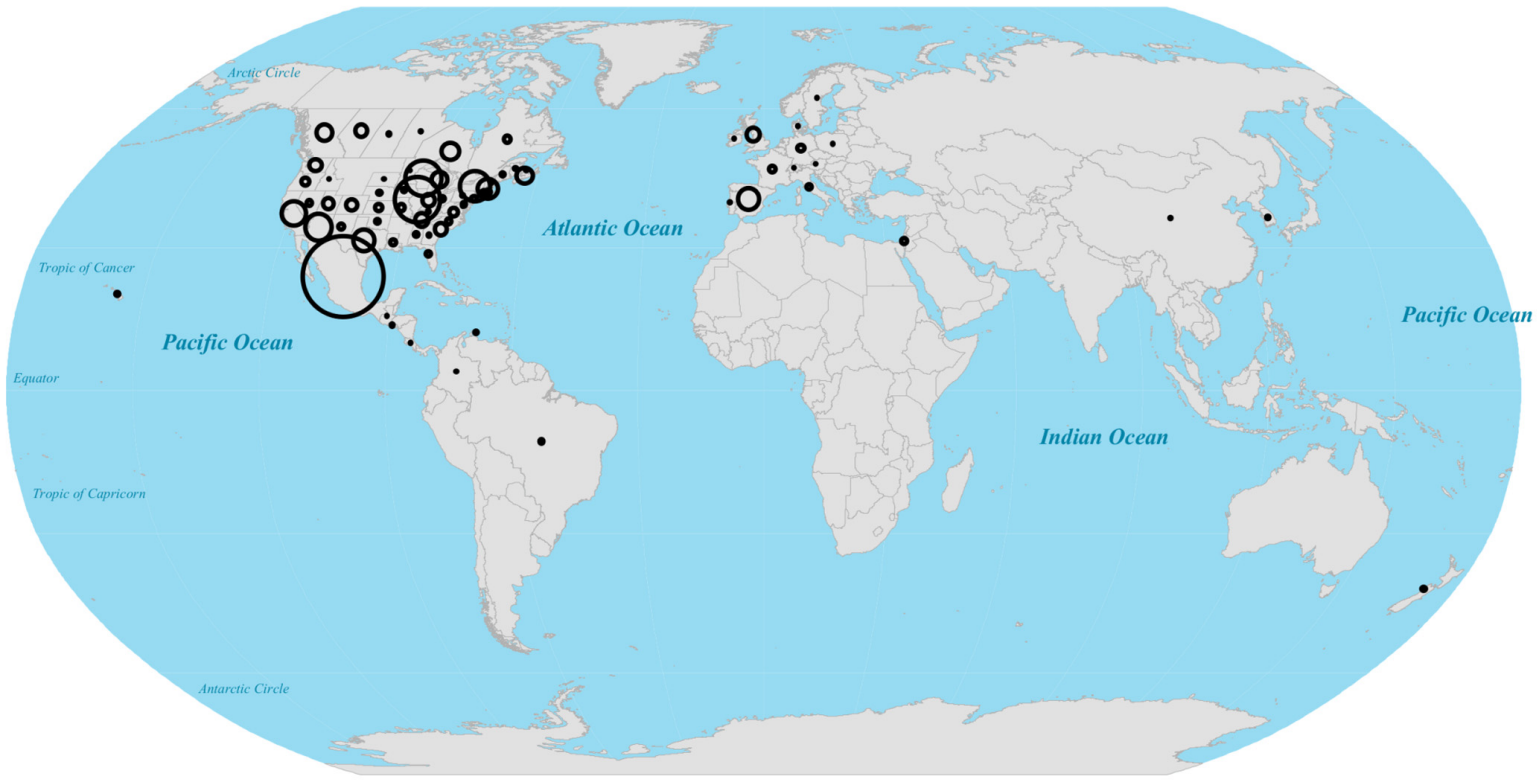
Global distribution of confirmed 2009 A/H1N1 influenza cases. Number and location of all confirmed human cases worldwide, as of May 8^th^, 2009.

**Figure 2:**
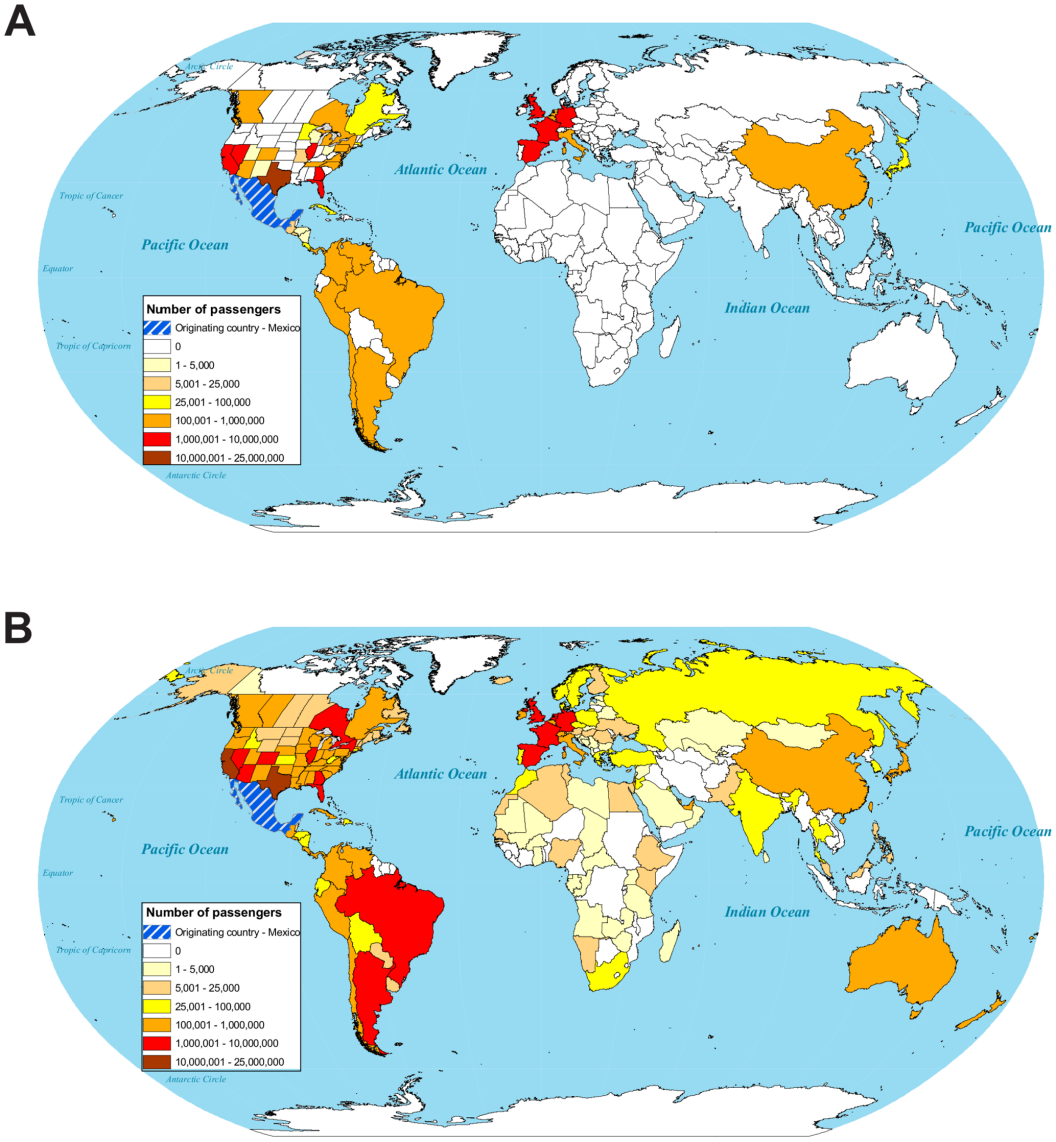
Global travel from Mexico in March–April 2009. (A) Estimated air travel (# passengers) directly from Mexico. (B) Direct flight plus estimated indirect air travel from Mexico.

As of May 8^th^ 2009, only two weeks after it was first reported, the 2009 A/H1N1 influenza strain had spread to 24 countries, 40 U.S. states (plus the District of Columbia) in the US, and 9 provinces in Canada (Figure 1). This rapid spread resulted, in part, from the tight connectivity of the globe through air travel (Figure 2).

A log-logistic survival analysis regression model was used to predict the time-to-reporting of the first confirmed 2009 A/H1N1 case to each country. Of all the models evaluated, a multivariate model with three predictors, (1) total country-level healthcare spending per capita, (2) estimated passenger volume arriving from Mexico via direct flights (direct flight capacity), and (3) passenger volume from Mexico via indirect, or two-leg, flights (indirect flight capacity), provided the best fit to the data using AIC, as detailed under Methods (Table 1, ΔAIC = 0, overall χ^2^ = 54.33 on 5 degrees of freedom, p-value<0.0001). The correlation between total country-level healthcare spending and the flight data was low (r<0.4). Although the correlation between direct and indirect flight data was high for countries with direct flights (r>0.9), the indirect flight information provided critical additional information for areas without direct flights. The AIC scores demonstrated this, as the model that included only direct flight information and healthcare spending did not explain the data as well as the best fit model (ΔAIC = 9.044). Alternate socio-economic measures, even those directly related to healthcare, such as the number of physicians per capita, GDP, or population density were much less predictive than total healthcare spending per capita. Notably, out of univariate analyses, the model with healthcare spending per capita as the sole predictor fit better than models with flight information alone (Table 1), demonstrating just how informative this data is in predicting the date of reporting. In the best fitting multivariate model, indirect flight capacity had the largest effect size, but including healthcare spending per capita substantially increased the fit to the data (Tables 1, 2). For Canadian provinces and American states, we conducted an analysis with just the flight data (Table 3 overall χ^2^ = 22.89 on 2 degrees of freedom, p-value <0.001). While the direct flight information does not have a statistically significant effect, the indirect does, most likely because only a few key hubs had direct flights, and these hubs also have a large volume of indirect connections.

**Table 1:** Akaike's Information Criterion, DAIC, and Akaike's weights of 14 survival analysis models, based on the Log-logistic survival time distributions, and the use of Gross Domestic Product (GDP), Healthcare Spending per Capita, Number of Physicians per Capita, Direct and indirect flights as predictors.

Model Predictors	AIC	ΔAIC	Akaike Weights
Direct and Indirect Flights, plus Healthcare Spending per Capita, including interaction effects	221.411	0.000	0.363
Direct and Indirect Flights, plus Healthcare Spending per Capita, and Population Density including interaction effects	221.986	0.574	0.273
Direct and Indirect Flights, plus Healthcare Spending per Capita, and GDP including interaction effects	222.943	1.532	0.169
Direct and Indirect Flights, plus Healthcare Spending per Capita, and GDP excluding interaction effects	223.740	2.329	0.113
Direct and Indirect Flights, plus Healthcare Spending per Capita excluding interaction effects	225.395	3.984	0.050
Direct and Indirect Flights, plus Healthcare Spending per Capita and Population Density excluding interaction effects	227.350	5.939	0.019
Direct and Indirect Flights, plus GDP including interaction effects	229.415	8.004	0.007
Direct and Indirect Flights, plus GDP and Number of Physicians including interaction effects	231.083	9.672	0.003
Direct and Indirect Flights, plus GDP and Number of Physicians excluding interaction effects	231.323	9.912	0.003
Healthspending per capita alone	234.226	12.815	0.001
Direct and Indirect Flights, plus Number of Physicians including interaction effects	235.086	13.675	0.000
Direct and Indirect Flights, plus Number of Physicians excluding interaction effects	235.138	13.727	0.000
Direct and Indirect Flights, plus GDP excluding interaction effects	236.222	14.811	0.000
Direct and Indirect Flights, plus Population Density and Number of Physicians including interaction effects	237.126	15.715	0.000
Direct and Indirect Flights alone	242.271	20.860	0.000
Direct and Indirect Flights, plus Population Density and Number of Physicians including interaction effects	242.613	21.201	0.000
Direct and Indirect Flights, plus Population Density excluding interaction effects	244.256	22.845	0.000
Direct and Indirect Flights, plus Population Density including interaction effects	244.469	23.057	0.000
GDP only	244.612	23.201	0.000
Direct Flights only	255.865	34.454	0.000
Number of Physicians only	255.913	34.502	0.000
Null Model	264.424	43.013	0.000
Population Density only	266.357	44.946	0.000

Interaction effects, when included, are only pairwise, for each set of flights and each socioeconomic factor (e.g., Healthcase Spending x Indirect Flights is used, but neither GDP x Healthcare Spending nor Direct x Indirect Flights is examined due to cross-correlation).

**Table 2:** Log logistic survival analysis regression of best fit model (ΔAIC = 0).

Coefficient	Coeff.	S.E.	p-value
Intercept	4.4540	0.0231	<0.0001
Direct Flights	−0.0057	0.2506	0.9818
Indirect Flights	−0.3605	0.1914	0.0596
Healthcare Spending per Capita (HSC)	−0.0371	0.0126	0.0033
Interaction of Direct Flights & HSC	−0.0833	0.1228	0.4975
Interaction of Indirect Flights & HSC	0.1775	0.1221	0.1460
Natural Logarithm of Scale parameter	−3.0862	0.1843	<0.0001

Best fit model has χ^2^ goodness of fit of 54.33 on 5 degrees of freedom, with a p-value <0.0001, on observations of 130 countries, 24 of which had confirmed cases. The interactions remain in the model because they improve the overall model fit based on AIC (c.f., Table 1).

**Table 3:** Log logistic survival analysis regression of a model of the predictive power of flight data for Canadian provinces and U.S. states.

Coefficient	Coeff.	S.E.	p-value
Intercept	4.3398	0.0048	<0.0001
Direct Flights	0.0039	0.0085	0.643
Indirect Flights	−0.0412	0.0089	<0.0001
Natural Logarithm of Scale parameter	−3.4829	0.1169	<0.0001

This model has χ^2^ goodness of fit of 22.89 on 2 degrees of freedom, with a p-value <0.0001, on observations of 11 Canadian provinces, 50 U.S. States, Puerto Rico, and the U.S. Virgin Islands, 51 of which had confirmed cases.

**Figure 3:**
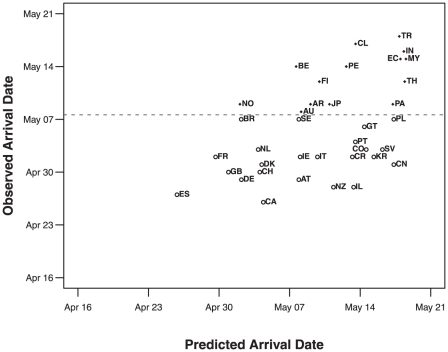
Model predictions compared with actual case detection dates. Open circles show predicted and observed detection dates for countries that reported H1N1 infections before our cut off of May 8th. Solid dots show the forward-prediction model validation of predicted and observed detection dates for countries that reported H1N1 infections after the cut off but before May 18th (see text for additional details). Country abbreviations are ISO 3166 two letter codes: AR: Argentina, AU: Austria, AL: Australia, BE: Belgium, BR: Brazil, CA: Canada, CH: China, CL: Chile, CO: Colombia, CR: Costa Rica, DE: Denmark, EC: Ecuador, ES: El Salvador, FI: Finland, FR: France, GE: Germany, GU: Guatemala, IN: India, IR: Ireland, IS: Israel, IT: Italy, JA: Japan, MA: Malaysia, MX: Mexico, NE: Netherlands, NO: Norway, NZ: New Zealand, PA: Panama, PE: Peru, PG: Portugal, PO: Poland, SK: South Korea, SP: Spain, SW: Sweden, SZ: Switzerland, TH: Thailand, TU: Turkey, UK: United Kingdom, US: United States.

For the country-level analysis, we compared the predicted reporting dates with the actual reporting dates, for countries where the disease arrived by May 8^th^, 2009 (Figure 3, Supplemental Online Figure S1). We validated the model by determining how well a model fit to data up until May 8th predicted reporting dates for fourteen countries where the disease was detected between May 9^th^ and May 19^th^ (Supplemental Online Figure S2). The correlation between forward predicted and observed dates was 0.62, and the observed reporting date fell within the 95% confidence interval for all countries. Many of the actual reporting dates are earlier than predicted, which is expected due to the non-linear nature a of log-log survival analysis regression. In particular, countries that had not reported disease by the cut-off date were included in the analysis by designating these as locations that “survived” the entire study period without acquiring the disease (i.e, censoring). This appropriately extends the predicted reporting dates by including information on both countries that had reported disease by the cut-off date as well as countries that had not. Using this methodology, we also estimated the reporting date of the disease in the remaining 103 countries and the 95% confidence intervals ranged from April 17^th^ to May 29^th^, 2009 (Supplemental Online Figure S3).

**Figure 4:**
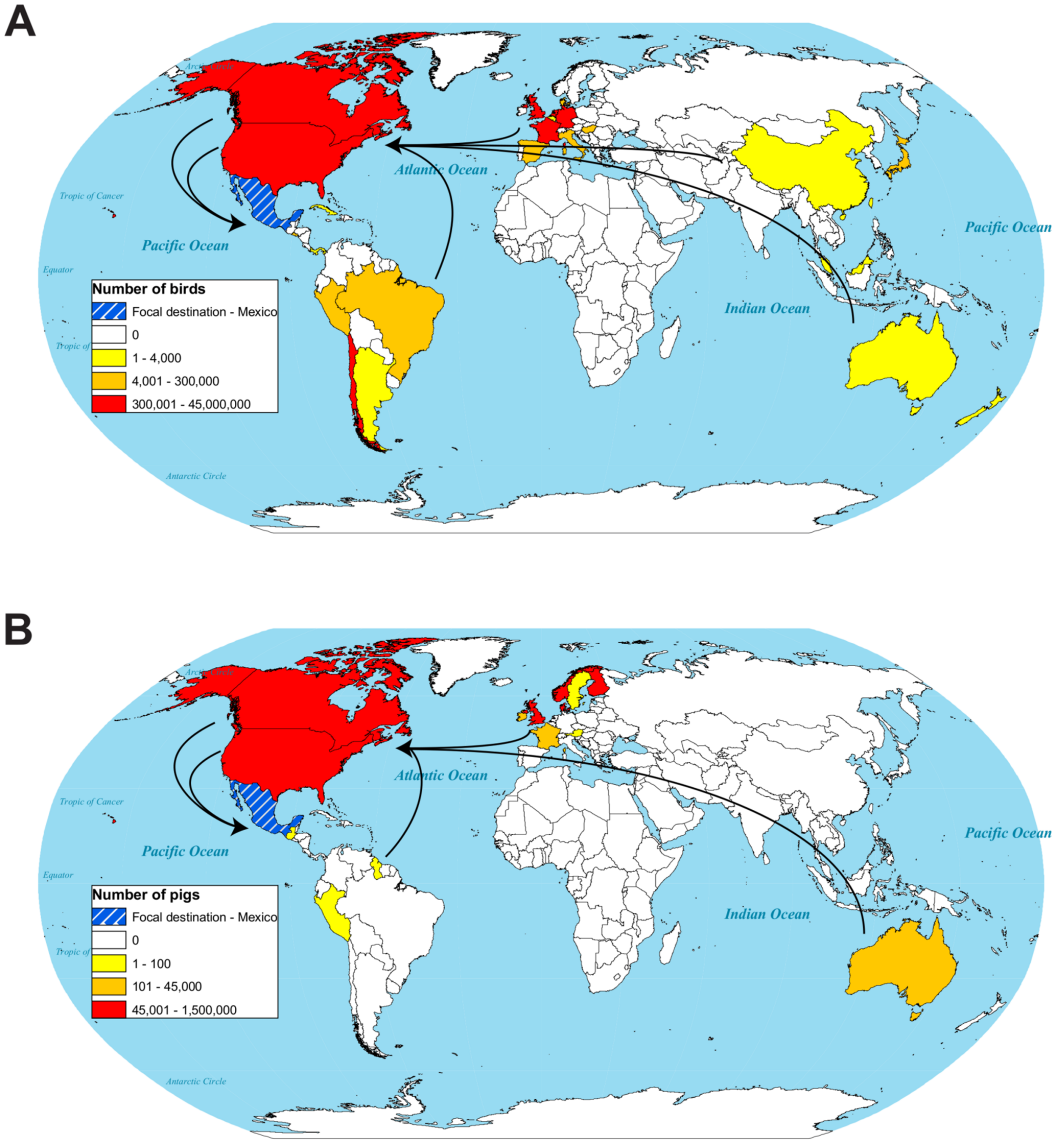
Global trade in live animals from 1998 through 2008. (A) Estimated number of live poultry traded, (B) Estimated number of live swine traded, internationally over the last decade, for Canada and the United States data are for trade directly to Mexico, for all other nations the data are for trade to Canada, Mexico and the United States, data from U.N.F.A.O.

To elucidate the potential origins of this novel viral strain, and to shed light on targets for future surveillance and prevention programs, we analyzed global trade in live poultry and swine during the decade preceding the current pandemic [14]. We estimate the trade in live swine between Canada, the United States and Mexico to be over 1.75 million animals over the last decade, while trade between North America and Eurasia is estimated to be over 750,000 animals (Figure 4b), The trade in live poultry is even larger, with Canada, the United States and Mexico trading over 41 million birds over the last decade, while trade between North America and Eurasia is estimated to be over 19 million birds (Figure 4a). Our results show that even though trade in live animals from Eurasia directly to Mexico has been minimal, there has been substantial movement of animals between Eurasia and the United States and Canada (Figure 4), coupled with substantial movement of animals from the United States and Canada into Mexico.

## Discussion

Previous studies suggest that data on air travel can be used to predict the spread of newly emerged human pathogens and better target public health measures [15], [16], [17], [18]. Our analyses support this, but demonstrate that the ability of a country to rapidly detect, diagnose, and report the new infection is a critical element that enhances our predictive power and control capacity. Other studies suggest that analysis of the underlying drivers of disease emergence (e.g. agricultural intensification, land-use change) can be used to predict the geographic origins of new emerging diseases[2]. The currently circulating pandemic influenza strain is a triple reassortment virus with closest known relatives from Europe, Asia, and North America, but there is uncertainty regarding its origin due to the large temporal separation between this pandemic 2009 A/H1N1 strain and the nearest ancestors (10–15 years) [7]. Our analyses of swine and poultry trade demonstrate an enormous potential for intercontinental mixing of potentially zoonotic pathogens, including influenza A viruses. Although artificial insemination is the predominant strategy for interbreeding of commercial swine, live swine are still routinely traded for breeding purposes [19]. Large numbers of poultry are also traded globally, and low pathogenicity influenza viruses are likely to spread unnoticed among poultry until they reassort or mutate to highly pathogenic forms, such as the A/H1N1v strain. This strain notably was the results of reassortment of several relatively low pathogenic influenza strains, as explained by Garten et al.[8]. In addition, as the recent cases of workers exposing a herd of pigs to the 2009 A/H1N1 virus makes clear [20], [21], even dramatic reductions in the international live animal trade may not prevent the exposure of local livestock to novel viral types from distant locations [9], [10].

Although extensive trade of poultry and swine between continents and within the North American countries almost certainly contributed to the emergence of this virus, surveillance of influenza strains circulating among traded animals is poor [10], so that it is impossible to designate any single country, trade connection or market as the key point at which the new strain evolved. Expanded surveillance for influenza in livestock populations may allow more of the markers of transmissibility and virulence to be identified, or factors driving higher virus transmission to be determined [9], [22]. In particular, we need to analyze all influenza strains, including the non- and low pathogenic influenzas, in addition to the highly pathogenic ones, with greater regularity. Only by this thorough surveillance can we begin to understand what differentiates the strains that cause pathogenesis in humans from those that do not. Such that eventually we may be able to predict viral emergence and develop vaccines against pandemic influenza viruses in advance of their spread. In order to develop such capability, we need to do more surveillance of livestock and wild influenza strains now.

The speed at which 2009 A/H1N1 spread during the early phases of this pandemic is striking. It was detected in four continents within three weeks after Mexican authorities first reported it. In contrast, the 1918 Spanish flu took 3 years to circle the globe [23]. Our analyses of air-travel data support the WHO's decision to recommend against closing all air travel from Mexico, since the virus most likely had already spread to several other countries by the time it was first reported to be widespread in Mexico on April 29^th^. In particular, cases had already been detected in the United States, which is a major hub for connecting flights [24].

Our current report is the first published analysis of H1N1 spread to include indirect flight data, and this significantly increased the predictive power of our model. Our analysis suggests that airports serving as major hubs could be targets for disease surveillance, and could become facilities that train people and stockpile medicines in preparation for pandemics. This approach differs from previous reports that focus on the role of travel restrictions at hubs [6], [17].

Our results further suggest a critical role for health care spending in determining a country's probability of detecting, confirming and reporting influenza cases in the early phases of a pandemic. The negative relationship between healthcare spending and detection of 2009 A/H1N1 influenza may be due to a delay in testing or in the collecting of specimens from individuals in countries lower healthcare resources. These countries likely have lower rates of health insurance, less healthcare infrastructure, lower self-reporting, and lower numbers of doctors per capita.

One consequence of lower health care resources is that the threshold for detection (i.e., the number of cases that need to occur before a case is detected, tested and confirmed by medical authorities) is likely higher in lower-income countries that cannot afford to invest as much in public health and healthcare infrastructure. Similar socioeconomic factors have been shown to play an important role in determining spatiotemporal patterns of diseases such as tuberculosis, schistosomiasis, West Nile virus, and HIV/AIDS [12], [13], [25], [26].

We found that incorporating data on healthcare spending per capita significantly increased our power to predict the time of reporting of 2009 A/H1N1. This suggests important strategies for future disease control. During the early stages of a pandemic, countries with moderate to high air travel from a pandemic origin, but relatively low healthcare spending, are likely to significantly under-report cases. It is therefore in the best global health interest for intergovernmental and other aid agencies to specifically target these nations for assistance to test and report cases early in a new pandemic. We propose that subsidies for outbreak response to these nations with high connectivity and low resources would be the most effective strategy to reduce the spread and impact of a pandemic.

Efforts to better target pandemics would be more effective in reducing disease spread if they were set up in advance of a pandemic [5], [6], [16], as there is a very small window of opportunity in which to act once a new emerging disease is detected. Such efforts could be strategically positioned to target emerging disease ‘hotspots’ [2] that are also hubs of trade and travel for surveillance and prevention [16], [27]. For influenza viruses, any future identification of a spillover of a novel strain from poultry or swine to farm workers should be rapidly followed by analyses of the travel routes out of the country where the index case was discovered. At that point, intergovernmental agencies such as WHO could best target limited resources to the poorer countries that are most likely to receive high numbers of airline travelers from the pandemic origin. These are the countries where reporting is likely to be poorest, and where a significant, undetected caseload is likely to exist by the time resources are allocated. These at-risk countries are also the least capable of affording control measures.

On the whole, this H1N1 strain appears to be relatively mild, although it is still inflicting additional morbidity and mortality. However, if a strain with a higher mortality rate, such as that observed with the H5N1 avian influenza subtype, were to spread in a similar fashion, the outcome would be catastrophic both in terms of human suffering and economic damage. For example, the impact of an H5N1 avian influenza outbreak, should the virus become easily transmissible between humans, on the United States economy has been estimated to be $71.3–$166.5 billion[28]. The measures we have proposed are likely to have economic benefits that far outweigh their costs.

## Methods

### Human Travel

We compiled the data on international air travel from the IATA database, supplied by Diio, LLC through their APGdat service[29]. Similar to prior analyses [15], [16], [17], [18], we used direct connection information with regards to aircraft type and passenger capacity to calculate the connectivity of Mexico with all airports included in the database, and summarized this information (as direct flight capacity) at the country level. Additionally, we estimated the number of connecting passengers (indirect flight capacity) by calculating the number of passengers (***p_i,j_***) arriving at airport ***j*** from airport ***i***, and then estimating the number of passengers (***p_j,k_***) going from airport ***j*** to airport ***k***, based on all flights reported in the database. We limited the potential connections (trip ***j→k***) to flights that departed no sooner than one hour after the first trip (***i→j***), and no later than six hours after the arrival of the first trip. We also disallowed return of passengers to Mexico once they left the country, and the return of passengers to North America once they left that region. We thus obtained a quantity, ***x_i,j,k_***, that estimates the total potential connections to airport ***k*** available to passengers from the first trip (***i→j***). Setting constant the fraction of all passengers that connect (***χ***), we obtained an estimate of the number of passengers with two leg itineraries for each potential destination (***i→k***; Eq. 1):
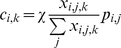
(1)


We summarized these connections at the country scale, thereby estimating connectivity for nearly every country on the globe with Mexico through either direct or indirect flights; the only countries excluded would require an overnight stay in a hub airport, or three or more connecting flights. We validated our algorithm (eq. 1) for connections within the contintental U.S.A. (the only data on actual itineraries, including connecting flight information, to which we had access). We randomly chose 50 connecting itineraries within the U.S.A. and compared our predictions to the actual routes. Our predictions were statistically significant, using a simple proportional model with log-normal errors, and explained over 60% of the variance in actual routes (F = 83.71, p<0.001 on 1, 49 d.f, adjusted R^2^ = 0.6232).

### Statistical Models

We determined the date a country reported its first WHO-confirmed 2009 A/H1N1 case through May 8^th^, 2009. We chose this date in order to limit the analysis, as much as possible, to initial spread from Mexico, because it served as a natural breakpoint in the distributions of reporting dates, as well as being the date our initial analysis. We performed a survival analysis using R [30], and used an accelerated life time model using a log-logistic distribution. We also examined using a scale-free exponential distribution, as opposed to a log-logistic distribution, which requires a scale parameter, but these models did not fit nearly as well, as measured by AIC. We followed Burnham and Anderson [31], in using Akaike Information Criterion (AIC) to choose the model that best explains the data (i.e., the one with the lowest AIC, or equivalently ΔAIC, score). Additionally we provided the Akaike weights, which estimate the likelihood that a specific model is the true model, assuming that the true model is in the set of examined models [14]. Using this methodology, we choose to evaluate 22 models that made mechanistic sense including a null model for a reference. We did not include any models with only the indirect flight data, and without the direct flight data, because we feel that this does not make mechanistic sense. To reduce multicollinearity we included at most two socio-economic indicators.

We evaluated four independent predictors for the date of first confirmed 2009 A/H1N1 case: the volume of (1) direct and (2) indirect passengers on international flights, (3) the country-specific Gross Domestic Product and (4) healthcare spending per capita, by both private and public entities, from 2006 (the most recent year with all data available) from World Bank estimates[32]. We also examined alternate socio-economic metrics as compiled by the World Bank[32], such as the number of physicians, and average population density. However models including these predictors did not perform as well (as measured by AIC) and often had many more missing values if limited to most recent information.

For all analyses, dates were transformed to Julian day since February 15^th^, and all predictor variables were standardized (mean subtracted, then divided by standard deviation) in order make possible the direct comparison of coefficients. This standardization has the added advantage of canceling out the ***χ*** factor in equation 1 for the statistical analysis; thus, our analyses do not require any assumptions about the number of passengers who make connecting flights.

These statistical models were used to predict the expected time of detection for all countries in our database that had GDP, population density, healthcare, and flight data. Confidence intervals were constructed from the best model fit based on the variance of the data, using the “predict” functions in R [30].

### Poultry and Swine Trade

We obtained United Nations Food and Agriculture Organization data on trade in Live Swine (commodity code HS96:S0103) and Live Poultry (S0105) from the U.N. Comtrade data portal[14]. We analyzed data from the last ten years (the approximate time since 2009 A/H1N1 diverged from the nearest sampled virus) [7], and focused on trade to North America (Mexico, Canada and United States) from outside this region, as well as trade to Mexico within the North American region.

## Supporting Information

Figure S1 Model predictions compared with actual case arrival dates. Dates of case arrivals (black diamonds) for cases that were reported before our cut off of May 8th. Grey whisker plots represent 95% confidence intervals for predicted arrival date, with interior grey bar as expected (mean) date of arrival from survival analysis.
(0.02 MB PDF)

Figure S2 Forward prediction of future case arrival dates. Dates of case arrivals (black diamonds) for cases that were reported after our cut off of May 8th, but before May 19th. Grey whisker plots represent 95% confidence intervals for predicted arrival date, with interior grey bar as expected (mean) date of arrival from survival analysis.
(0.02 MB PDF)

Figure S3 Forward prediction of future case arrival dates. Grey whisker plots represent 95% confidence intervals for predicted arrival date, with interior grey bar as expected (mean) date of arrival from survival analysis.
(0.03 MB PDF)
