# Supplementary figures and images for: Predictive Power of Air Travel and Socio-Economic Data for Early Pandemic Spread
Source: PLoS One. 2010 Sep 15;5(9):e12763. doi: 10.1371/journal.pone.0012763 (PMC2939898; doi:10.1371/journal.pone.0012763)

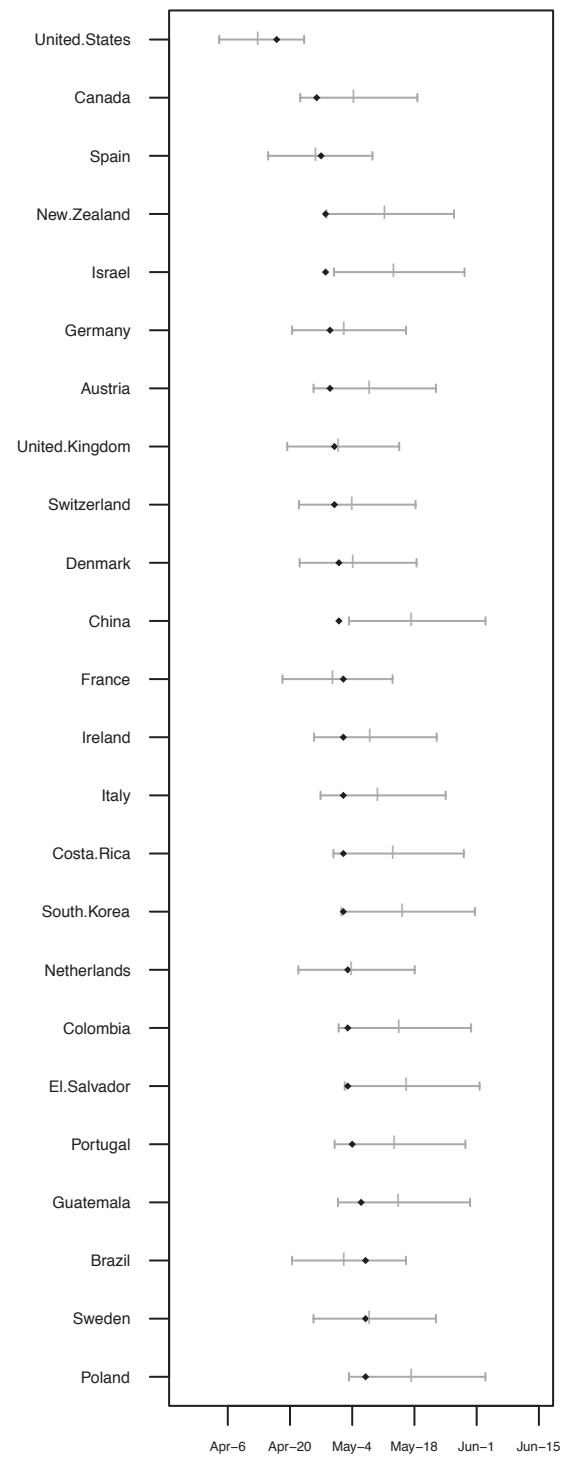

Supplement: Figure S1 — Model predictions compared with actual case arrival dates. Dates of case arrivals (black diamonds) for cases that were reported before our cut off of May 8th. Grey whisker plots represent 95% confidence intervals for predicted arrival date, with interior grey bar as expected (mean) date of arrival from survival analysis. (0.02 MB PDF) [file pone.0012763.s001.pdf]

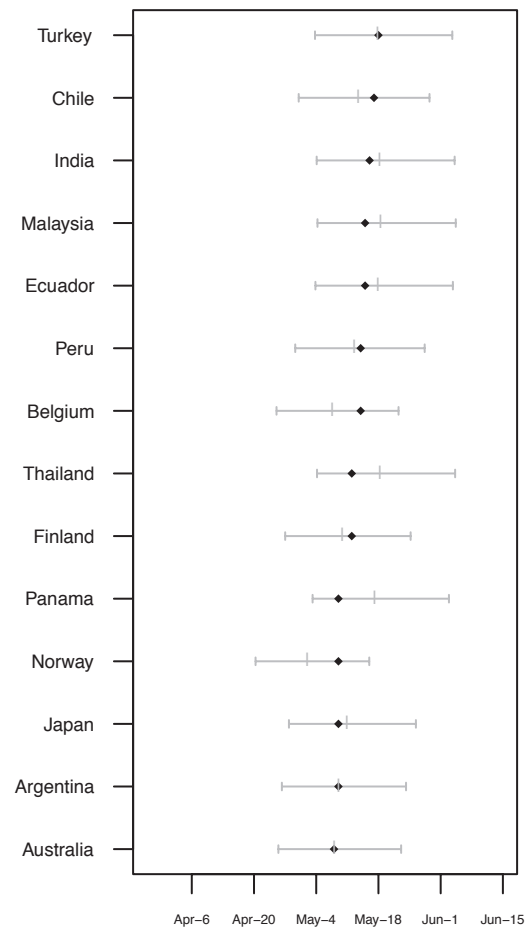

Supplement: Figure S2 — Forward prediction of future case arrival dates. Dates of case arrivals (black diamonds) for cases that were reported after our cut off of May 8th, but before May 19th. Grey whisker plots represent 95% confidence intervals for predicted arrival date, with interior grey bar as expected (mean) date of arrival from survival analysis. (0.02 MB PDF) [file pone.0012763.s002.pdf]

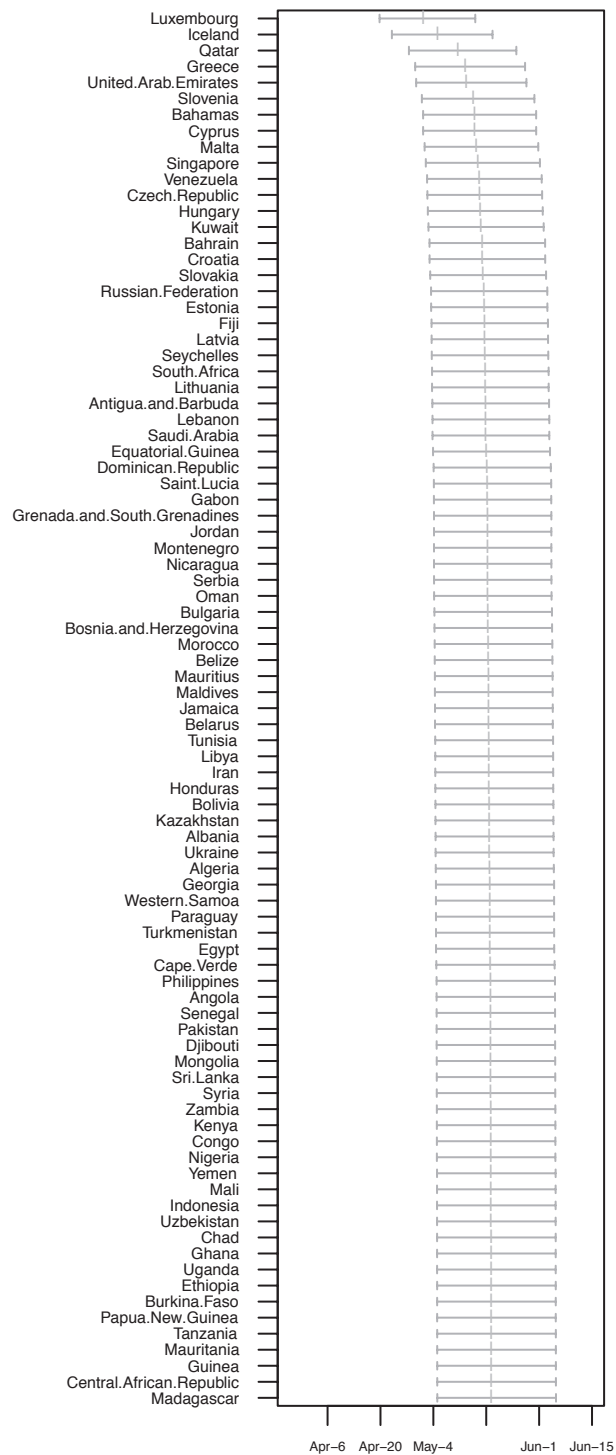

Supplement: Figure S3 — Forward prediction of future case arrival dates. Grey whisker plots represent 95% confidence intervals for predicted arrival date, with interior grey bar as expected (mean) date of arrival from survival analysis. (0.03 MB PDF) [file pone.0012763.s003.pdf]
